# Supplementary material for: Efficacy of a smartphone app to improve mental health among emergency service workers: A randomised controlled trial
Source: PLoS One. 2026 Feb 5;21(2):e0342419. doi: 10.1371/journal.pone.0342419 (PMC12875461; doi:10.1371/journal.pone.0342419)
Supplement: S1 File — (DOCX) [file pone.0342419.s001.docx]

## Supplement 1. App content

Table S1_A. Intervention activities

| **MINDFULNESS** |
| --- |
| - Responding mindfully - *Learn how to manage stress and distress by living more in the present moment.* (video) - Mindfulness (8x audio) |
| **MANAGING THOUGHTS** |
| - Managing thoughts - *Understand how thoughts, feelings, and behaviour are connected and how to manage them. (video)* - Lighten the load *– Unload unpleasant thoughts through acceptance and defusion* - Notice your thinking traps *– Identify and label unhelpful thinking styles* - Worry time *– Get intrusive thoughts out of your head to manage them more effectively.* - Gratitude diary *– Develop a daily gratitude practice to improve wellbeing and mood.* |
| **HEALTHY COPING** |
| - Healthy coping – *Develop a plan for healthy coping with stress and distress. (video)* - What is Trauma? *– Find out how to deal with trauma and understand the signs & symptoms?  (video)* - Tension Release *– Reduce tension and strain brought on by stress and anxiety. (audio)* - Understanding warning signs *– Know what to look out for so you to stay on track.* - Breathe *– A quick ground tool to recenter when you feel anger, panic, or distress that is hard to manage.* - Getting active *– Improve mood and relieve stress by getting more physical activity.* - Sleep sounds *– A brief exercise to aid sleep. (audio)* - Rethinking your drinking *– Develop healthier drinking habits.* - Score your sleep *– Tips to improve your sleeping habits.* - Problem solver *– A useful guide to working through problems when you're struggling with unhelpful worries.* - 5-things (taking notice) – *Reconnect with your physical space when you are caught up with intrusive thoughts or heightened emotion. (audio)* |
| **VALUED ACTION** |
| - Value driven action *– Improve your mood by recognising and integrating what truly matters to you into your life. (video)* - Connect with others *– Improve low mood through connecting to others.* - Working Out Your Values *– Identify and prioritise life values for importance.* - Values Bootcamp *– Plan goals for specific value areas.* - Being Assertive *– Improve your communication style for better relationships.* - Supporting others *– Help support those you care about who might be struggling.* |


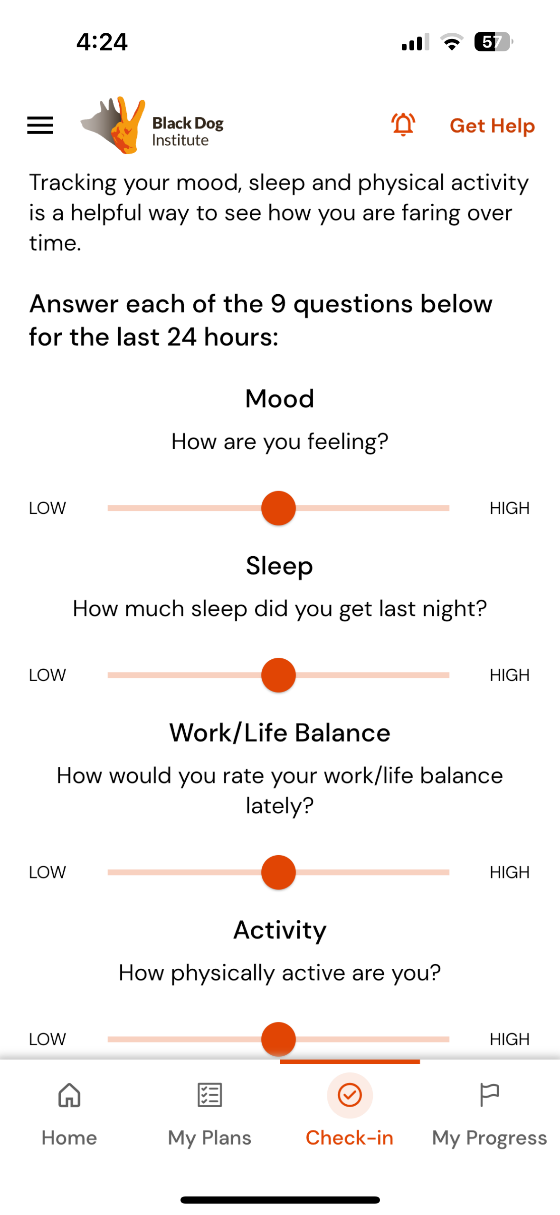

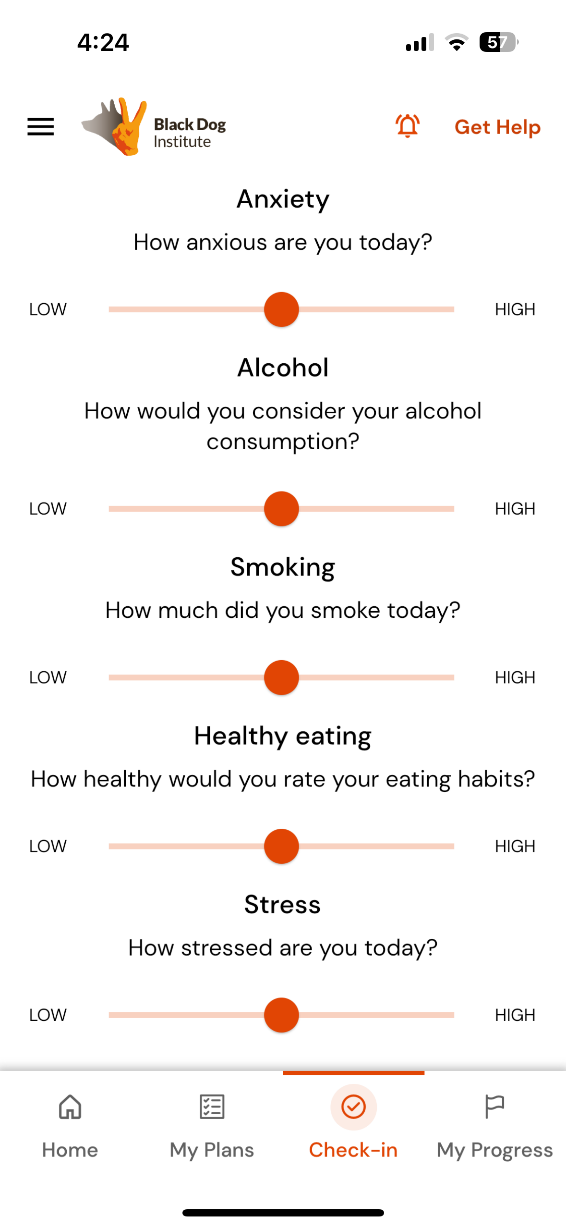


Figure S1_A. monitoring items
