# Supplementary material for: Efficacy of a smartphone app to improve mental health among emergency service workers: A randomised controlled trial
Source: PLoS One. 2026 Feb 5;21(2):e0342419. doi: 10.1371/journal.pone.0342419 (PMC12875461; doi:10.1371/journal.pone.0342419)
Supplement: S2 File — (DOCX) [file pone.0342419.s002.docx]

## Supplement 2. User Engagement items

|  | **Not at all** |  | **Somewhat** |  | **Completely** |
| --- | --- | --- | --- | --- | --- |
| 1. How well did you understand the content of the app? | 1 | 2 | 3 | 4 | 5 |
| 1. How easy is the app to use? | 1 | 2 | 3 | 4 | 5 |
| 1. Is the app content (visual information, language, design) appropriate for you? | 1 | 2 | 3 | 4 | 5 |
| 1. Is the app interesting/engaging? | 1 | 2 | 3 | 4 | 5 |
| 1. Do you feel that the app has helped you improve your mental fitness? | 1 | 2 | 3 | 4 | 5 |
|  | **Not at all** |  | **Maybe** |  | **Definitely** |
| 1. Would you recommend this app? | 1 | 2 | 3 | 4 | 5 |
|  | **Low** |  |  |  | **High** |
| 1. What is your overall rating of the app? | 1 | 2 | 3 | 4 | 5 |
|  |  |  |  |  |  |

1. What feature of the app did you like most? ___________________________________________________________________________
2. What feature of the app did you like the less? ___________________________________________________________________________
3. If you stopped using the app, why did you do so?
4. I did not stop before completing the 30-day trial
5. I didn’t have time
6. I lost interest
7. I didn’t like the app
8. I already got what I wanted/needed from the app
9. The app was not relevant to me
10. Technical issues/bugs
11. Other reason/s (please specify): _____________________________________________

1. Are there any features that you would like to see added to the app? ___________________________________________________________________________
