# Supplementary material for: Efficacy of a smartphone app to improve mental health among emergency service workers: A randomised controlled trial
Source: PLoS One. 2026 Feb 5;21(2):e0342419. doi: 10.1371/journal.pone.0342419 (PMC12875461; doi:10.1371/journal.pone.0342419)
Supplement: S3 File — (DOCX) [file pone.0342419.s003.docx]

## Supplement 3. Moderation for medication use and help-seeking

Exploratory analyses indicated primary outcome Time x Conditions were not moderated by taking medication for a mental health issue at trial registration (Bs ranged from 1.10 to 1.18, all |ts| < 1.10, all ps > .273). However, these Time x Condition effects were moderated by help-seeking in the four weeks before trial registration (baseline to 1-month three-way interaction: B = -3.30, 95% CI [-5.59, -1.01], t(577.82) = -2.84, p = .005; baseline to 3-month three-way interaction: B = -3.07, 95% CI [-5.14, -1.00], t(604.52) = -2.91, p = .004).

There was a different moderation pattern for the two time periods examined. For the period from baseline to 1-month, there was no significant Time x Condition interaction for participants who did help-seek (B = -1.64, 95% CI [-3.35, 0.06], t(251.63) = -1.89, p = .060). However, there was a significant Time x Condition interaction for participants who did not help-seek in the four weeks before trial registration (B = 1.61, 95% CI [0.06, 3.14], t(328.16) = 2.05, p = .041), reflecting a significant decrease in K10 scores from baseline to 1-month for the full intervention condition (B = -1.50, 95% CI [-2.71, -0.29], t(336.33) = -2.44, p = .015, d = -0.24), and an even greater significant decrease in K10 scores from baseline to 1-month for the tracking-only condition (B = -3.11, 95% CI [-4.06, -2.16], t(315.33) = -6.43, p < .001, d = -0.50). The difference in K10 scores at 1-month favouring tracking-only was significant (B = 1.82, 95% CI [0.20, 3.44], t(644) = 2.20, p = .028, d = 0.27).

For baseline to 3-months, there was no significant Time x Condition interaction for participants who did not help-seek (B = 1.02, 95% CI [-0.35, 2.40], t(345) = 1.47, p = .143). However, there was a significant Time x Condition interaction for participants who did help-seek in the four weeks before trial registration (B = -1.98, 95% CI [-3.55, -0.42], t(262) = -2.49, p = .013), reflecting a significant decrease in K10 scores from baseline to 3-month for the full intervention condition (B = -3.31, 95% CI [-4.98, -1.64], t(253) = -3.91, p < .001, d = -0.53), but not the tracking-only condition (B = -0.99, 95% CI [-2.40, 0.42], t(247) = -1.38, p = .169, d = -0.16). The difference in K10 scores at 3-months was not significant (B = -1.80, 95% CI [-4.12, 0.53], t(445) = -1.52, p = .130, d = -0.25).
