# Supplementary material for: Efficacy of a smartphone app to improve mental health among emergency service workers: A randomised controlled trial
Source: PLoS One. 2026 Feb 5;21(2):e0342419. doi: 10.1371/journal.pone.0342419 (PMC12875461; doi:10.1371/journal.pone.0342419)
Supplement: S4 File — (DOCX) [file pone.0342419.s004.docx]

## Supplement 4. App user ratings

***

***

**

***

** *p* <.01

*** *p* < .001

Figure S4_A. Comparison of mean app user ratings between intervention arms (error bars represent ±1 SD). Higher scores indicate more favourable app rating.
