# Supplementary material for: Efficacy of a smartphone app to improve mental health among emergency service workers: A randomised controlled trial
Source: PLoS One. 2026 Feb 5;21(2):e0342419. doi: 10.1371/journal.pone.0342419 (PMC12875461; doi:10.1371/journal.pone.0342419)
Supplement: S6 File — (DOCX) [file pone.0342419.s006.docx]

## Supplement 6. Intervention group characteristics at baseline, split by engagement level

|  | **Non-engagers**  **(n=229)** | **Minimal engagers**  **(n=151)** | **Engagers**  **(n=60)** |
| --- | --- | --- | --- |
| Age in years, M (SD) | 47.06 (12.86) | 42.94 (12.94) | 46.57 (13.84) |
| Gender identity, n (%)^a^ |  |  |  |
| Female | 64 (27.9) | 66 (43.7) | 30 (50.0) |
| Male | 157 (68.6) | 80 (53.0) | 30 (50.0) |
| Non-binary or other | 8 (3.5) | 2 (1.3) | 0 (0.0) |
| Education, n (%) |  |  |  |
| Year 12 (equivalent) or less | 35 (15.8) | 19 (13.1) | 13 (22.0) |
| Trade or other certificate or diploma | 94 (42.5) | 68 (46.9) | 17 (28.8) |
| University Degree | 92 (41.6) | 58 (40.0) | 29 (49.2) |
| Other | 8 (3.5) | 6 (4.0) | 1 (1.7) |
| Live in metropolitan area, n (%) | 69 (30.1) | 45 (29.8) | 18 (30.0) |
| Emergency service worker status, n (%)^b^ |  |  |  |
| Current paid | 129 (56.3) | 85 (56.3) | 22 (36.7) |
| Current volunteer | 73 (31.9) | 53 (35.1) | 31 (51.7) |
| Retired | 25 (10.9) | 13 (8.6) | 7 (11.7) |
| Service length, n (%)^c^ |  |  |  |
| < 1 year | 4 (1.7) | 3 (2.0) | 0 (0.0) |
| 1-5 years | 54 (23.6) | 47 (31.1) | 17 (28.3) |
| 6-10 years | 35 (15.3) | 19 (12.6) | 10 (16.7) |
| 10+ years | 136 (59.4) | 81 (53.6) | 33 (55.0) |
| Sought professional/clinical help in lifetime (mental health professional, GP), n (%) | 186 (81.2) | 124 (82.1) | 46 (76.7) |
| Engaged in help-seeking in last four weeks, n (%) | 83 (39.0) | 62 (42.5) | 29 (52.7) |
| Currently taking medication for a mental health issue*,* n (%) | 75 (32.8) | 46 (30.5) | 17 (28.3) |
| Experienced traumatic event, n (%) | 207 (90.4) | 131 (86.8) | 53 (88.3) |
| **Primary outcome** |  |  |  |
| K10, M (SD) | 25.79 (6.14) | 25.20 (6.27) | 25.68 (6.62) |
| **Secondary outcomes** |  |  |  |
| PHQ-9, M (SD) | 9.41 (5.18) | 10.03 (5.70) | 10.65 (5.76) |
| GAD-7, M (SD) | 7.33 (4.52) | 7.44 (4.54) | 7.64 (4.59) |
| PTSD-8, M (SD)^d^ | 15.99 (5.80) | 16.87 (5.32) | 17.38 (5.34) |
| CD-RISC-10, M (SD) | 26.99 (7.03) | 26.70 (6.95) | 27.23 (7.10) |
| CSE-T, M (SD) | 42.82 (11.14) | 43.77 (10.59) | 43.78 (12.42) |
| WHO-5, M (SD) | 38.27 (20.30) | 38.33 (21.28) | 37.07 (19.29) |
| AUDIT-C, M (SD) | 4.93 (2.82) | 3.75 (2.02) | 3.43 (2.26) |
| AQoL-4D, M (SD) | 77.67 (10.96) | 79.67 (10.24) | 77.64 (10.62) |
| Independent living | 95.58 (10.63) | 96.10 (9.62) | 96.85 (9.39) |
| Relationships | 68.70 (20.87) | 71.74 (19.91) | 68.52 (18.88) |
| Physical senses | 87.09 (11.89) | 89.04 (10.50) | 87.22 (12.24) |
| Mental health | 59.29 (18.16) | 61.81 (18.08) | 57.96 (17.84) |
| HPQ, M (SD)^e^ | 18.19 (5.74) | 18.43 (5.41) | 16.99 (6.26) |

^a^ Three participants in the minimal engager group indicated ‘prefer not to say’.

^b^ Two participants in the non-engager group indicated ‘prefer not to say’.

^c^ One participant in the minimal engager group indicated ‘prefer not to say’.

^d^ Administered only to participants who indicated that they experienced, witnessed or were confronted with a stressful experience or traumatic event in the last month.

^e^ Composite measure of effective workdays was calculated by multiplying work performance score for days worked during the previous 28 days by the number of days present at work over the same period.

Note. K10 = Kessler Psychological Distress Scale; PHQ-9 = Patient Health Questionnaire-9; GAD-7 = Generalized Anxiety Disorder-7; PTSD-8 = Post-Traumatic Stress Disorder 8-item; CD-RISC-10 = 10-item Connor-Davidson Resilience Scale; CSE-T = Trauma Coping Self-Efficacy Scale; WHO-5 = World Health Organization Wellbeing Index; AUDIT-C = Alcohol Use Disorders Identification Test-Concise; AQoL-4D = Assessment of Quality of Life 4-dimension version; HPQ = Health and Work Performance Questionnaire.
